# Graphene Oxide-Coated Gold Nanorods: Synthesis and Applications

**DOI:** 10.3390/nano10112149

**Published:** 2020-10-28

**Authors:** Thabang C. Lebepe, Sundararajan Parani, Oluwatobi S. Oluwafemi

**Affiliations:** 1 Department of Chemical Sciences, University of Johannesburg, P.O. Box 17011, Doornfontein, Johannesburg 2028, South Africa; calvyn.tl@gmail.com (T.C.L.); sbarani416@gmail.com (S.P.); 2 Centre for Nanomaterials Science Research, University of Johannesburg, Johannesburg 2028, South Africa

**Keywords:** gold nanorods, graphene oxide, biocompatibility, photothermal, sensor, theranostic

## Abstract

The application of gold nanorods (AuNRs) and graphene oxide (GO) has been widely studied due to their unique properties. Although each material has its own challenges, their combination produces an exceptional material for many applications such as sensor, therapeutics, and many others. This review covers the progress made so far in the synthesis and application of GO-coated AuNRs (GO–AuNRs). Initially, it highlights different methods of synthesizing AuNRs and GO followed by two approaches (ex situ and in situ approaches) of coating AuNRs with GO. In addition, the properties of GO–AuNRs composite such as biocompatibility, photothermal profiling, and their various applications, which include photothermal therapy, theranostic, sensor, and other applications of GO–AuNRs are also discussed. The review concludes with challenges associated with GO–AuNRs and future perspectives.

## 1. Introduction

**Figure 1:**
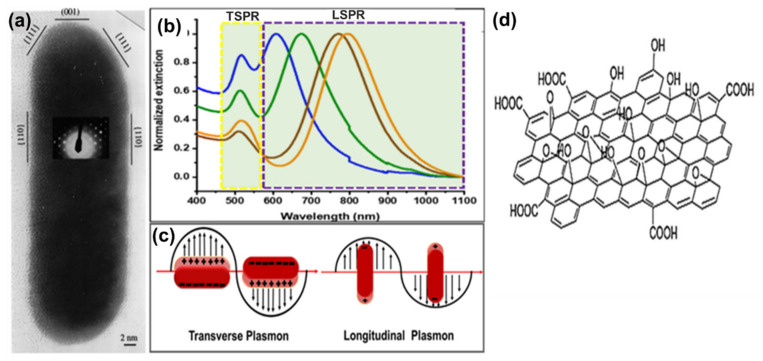
(**a**) High-resolution TEM image of gold nanorods (AuNRs) oriented along the (110) direction, showing the faceted crystal structure of the rod; the inset is the electron diffraction pattern recorded from the rod, which proves the single crystalline structure of the rod. Adapted from ref [18], with permission from Elsevier, 1999. (**b**) UV−Vis−NIR spectra of AuNRs with different transverse surface plasmon resonance (TSPR) and TSPR wavelength position. Adapted from ref [19], with permission from American Chemical Society, 2018. (**c**) Diagram illustrating the conduction band electron oscillation upon transverse (**left**) and longitudinal (**right**) localized surface plasmon resonances of AuNRs. (**d**) Typical structure of graphene oxide Adapted from [20] with permission from RSC advances, 2011.

Gold nanomaterials have received great attention because of their fascinating optical and electronic properties as well as their ability to be tuned in various absorption wavelengths for many applications [1,2]. Various architectures of gold nanomaterials such as nanoshells, nanostars, nanorods, nanospheres, and nanocages have been studied for the past two decades due to their unique localized surface plasmon resonance [1,3,4,5,6,7]. The localized surface plasmon resonance is an optical phenomenon that occurs due to the interaction between conduction band surface electrons and the incident light resulting in scattering and absorption phenomenon. This phenomenon is driven by the nanomaterial physical dimensions and medium of dispersion [8,9,10]. The anisotropic gold nanoparticles are more superior due to their structural, optical, electronic, magnetic, and catalytic properties compared to the nanospherical particles [11]. These anisotropic gold nanoparticles such gold nanostars and gold nanotriangles have been reported to absorb light in a wider spectrum [8]. Among these anisotropic gold nanoparticles, gold nanorods (AuNRs) can be produced and easily tuned to absorb light at different spectral regions by the simple manipulation of their aspect ratio (length/width ratio) [2,12,13]. The aspect ratio is responsible for the optical extinction spectrum in AuNRs [2,4]. Typically, AuNRs are described as particles with an aspect ratio between 2 and 25. AuNRs are single-crystalline, which orient in either the {100} or {111} or {110} directions, as shown in transmission electron microscopy images (Figure 1a) [14]. AuNRs produce the longitudinal surface plasmon resonance (LSPR) and transverse surface plasmon resonance (TSPR) in the absorption spectroscopy (Figure 1b) [14,15,16]. TSPR occurs due to the oscillation of free surface electrons along the width of the rod, which is usually observed in the visible region around λ_abs_ = 510–530 nm. TSPR show a little dependence on the aspect ratio of AuNRs (Figure 1c left). On the contrary, LSPR occurs due to oscillation along the length of the rod and is observed in the near-infrared (NIR) region. LSPR strongly depends on the AR. The higher the aspect ratio, the longer the absorption wavelength (Figure 1c right). AuNRs are also highly sensitive to local environmental changes, making them excellent materials for many applications [9]. Biomedical applications of AuNRs focus mainly on their potential to deliver/control release drugs and their therapeutic action against cancer, bacteria, and viruses [7,17]. In addition, AuNRs have also been used in other fields such as optical power limiters, solar cells, light-emitting diodes, stress/strain sensors, and catalysis [2,4].

Graphene oxide (GO) is a single atomic layer thick two-dimensional material, which is a derivative of graphene with various oxygen-containing functionalities such as epoxides, hydroxyl groups, and carboxyl groups [21] (Figure 1d). Unlike hydrophobic graphene, GO is hydrophilic due to the oxygen functionalities and hence, it is soluble in water [22]. GO has a honeycomb carbon structure with many defects localized inside as well as over the surface planes and edges induced by oxidation [23]. The structural properties of GO make it an ideal material of choice in the field of biomedicine, especially theranostic, due to its solubility in aqueous mediums, better colloidal stability, cost-effectiveness, scalability, and large surface area (≈2630 m^2^/g) and thermal property mostly in the reduced form [24,25,26]. GO has an exceptional capability to immobilize a huge quantity of substances, including metals, drugs, biomolecules, and fluorescent probes and cells [27]. Moreover, it has been reported to have intrinsic and NIR absorbance properties, which make it useful as a photothermal agent for cancer treatment [21,25,26,28]. The combination of GO and AuNRs (GO–AuNRs) advances the applications of this composite material in theranostic [29,30,31,32,33,34,35,36], biocarrier [30,36], imaging [32,37,38,39], and also sensors [40,41,42,43,44,45]. In this review, we discussed the composites of GO–AuNRs, including their different synthesis procedures, properties, and different applications. We first look at the synthesis of AuNRs and GO separately followed by a combination of both materials by different approaches.

## 2. Gold Nanorods Synthesis

**Figure 2:**
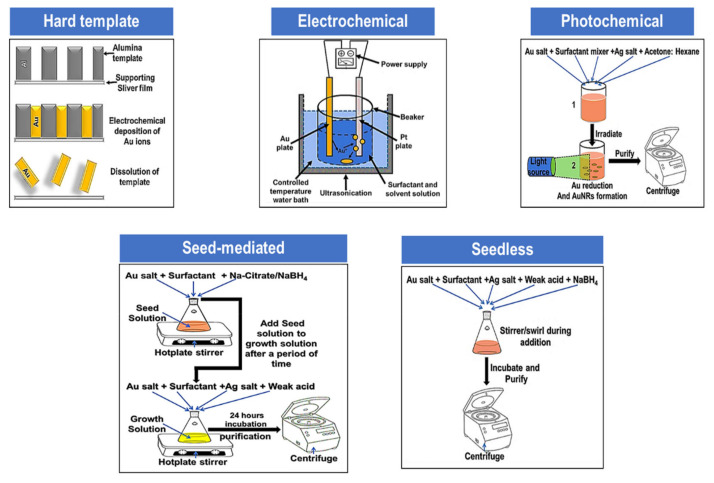
Schematic diagram of different AuNRs synthetic methods.

Since the discovery of AuNRs, several methods have been developed to solve the challenges in the synthesis, which includes high product yield, robustness to minor impurities, precise control over AuNRs surface chemistry, stability, and most importantly, a feasible method to suit specific applications [16]. Different methods such as the template method [46,47,48], electrochemical method, photochemical method [49,50], and seed-mediated method [4,13,51,52,53] have been reported for the synthesis of AuNRs [9] (Figure 2). A combination of some of these methods can also be carried out for advanced synthesis. Factors such as temperature [15,54], pH [19,53,55,56,57], type of surfactant [13,14,17,52,53,57,58,59,60,61], reagents concentration [10,16,50,62,63,64,65,66], additives [67,68,69], and the seed quality [4,51,52,53,70,71] influence the growth and purity of AuNRs.

### 2.1. Hard Template Method

The hard template method was introduced by Martin and co-workers where AuNRs were prepared by the electrochemical reduction of gold (Au) salt within the pores of nanoporous polycarbonate/alumina template membranes [5,16,46,47,49,72]. Briefly, the method is initiated by sputtering a small amount of silver ions or copper ions onto the alumina template membrane to provide a conductive film for electrodeposition. Thereafter, Au ions were electrodeposited within the nanopores of alumina, forming rods. The alumina template membrane and the copper or silver film are selectively dissolved in the presence of a polymeric stabilizer such as poly(vinylpyrrolidone) (PVP) and cetyltrimethylammonium bromide (CTAB). Finally, the rods were dispersed either in water or in organic solvents by means of sonication or agitation [16]. Even though the yield and uniformity of the AuNRs attained by means of template methods is high, it has some limitations, such as a complex method of releasing the rods from the template and dispersing them into solvents. Moreover, the AuNRs typically have moderately large diameters (>≈100 nm), which affect their plasmonic responses due to the hindrance effect [5,16,46,47,49,72].

### 2.2. Electrochemical Method

The electrochemical route to synthesize AuNRs comes by modification of the previous studies on the electrochemical synthesis of transition metal clusters through reverse micelles in organic solvent systems. This method also gives a high yield of AuNRs; however, this method requires a high cost to be deplored [5,14,16,17,49,50,73,74,75,76,77]. In short, the Au metal plate was used as a sacrificial anode, whilst the cathode was a platinum plate behind which a silver plate was placed, as it played an important role in controlling the aspect ratio of the rods. All the electrodes were immersed in an electrolytic solution containing a cationic binary surfactant cocktail of hexadecyltrimethylammonium bromide (CTAB) and tetradodecylammonium bromide (TC_12_AB). In this electrochemical reaction, CTAB acted as a stabilizer to prevent aggregation, while TC_12_AB acted as a rod-inducing co-surfactant. In addition, acetone was added to loosen the micellar framework, which facilitated the incorporation of a cylindrical-shape-inducing co-surfactant into the CTAB micelles, while cyclohexane was added to enhance the formation of elongated rod-like CTAB micelles. Then, the mixed solution electrolytic cell was placed inside an ultrasonic bath at 36 °C. Sonication was needed to dissipate the AuNRs and keep them away from the cathode during the electrolysis. During electrolysis, a current of 3 mA was run throughout for 30 min. The full mechanism of AuNRs formation by this method remains unclear. However, some researchers have reported that during the synthesis, the Au anode was consumed to form AuBr_4_^−^ anions, which were complexed to the surfactants followed by migration to the cathode, where reduction occurs [10,16,78].

### 2.3. Photochemical Method

Photochemical synthesis is similar to the electrochemical method, except that rods are formed using UV light as a reducing agent. In a typical experiment, a growth solution containing gold salt, CTAB and TC_12_AB surfactants, silver nitrate, acetone, and hexane was prepared, followed by irradiation of the solution with ultraviolet (UV) light at a wavelength of 254 or 300 nm depending on the aspect ratio required. Typically, 254 nm UV light was used to synthesize AuNRs that have LSPR in a wavelength range of 600–800 nm depending on the silver concentration in the growth solution, while 300 nm UV light produced LSPR in a wavelength range that is more than 800 nm. This method was time and energy consuming to obtain AuNRs when compared to the seed-mediated method [10,78].

### 2.4. Seed-Mediated Growth Method

The seed-mediated growth of the AuNRs method approach dates back to the early 2000s, whereby the stepwise-growth method was developed by controlling and modifying the reaction environment [4,10,49,60,63]. There are two types of preparation methods in a seed-mediated method based on the absence and presence of silver ions. The first method usually occurs in the absence of silver ions and normally produces decahedral gold nuclei, which grow into nanorods in the presence of CTAB. However, the yield under this method is usually low. The second method involves both silver nitrate and CTAB, where the Au seeds grow into a single crystal and rod-shaped particles with a high yield of nanorods. However, in this method, only ∼15% of the Au^3+^ is converted into Au^0^ [49,79]. The second method was further expanded by a combination of surfactants in the growth solution. This method was invented to reduce the standard CTAB (0.1 M) concentration, which was shown to be toxic when the AuNRs were applied in biological applications [49,53,57,79]. The seed-mediated methods required less energy as compared to electrochemical and photochemical methods.

#### 2.4.1. Seed-Mediated Method without AgNO_3_

The seed-mediated method without AgNO_3_ was proposed by Murphy and co-workers [13,58,63] by using different sizes of gold seed particles prepared from sodium borohydride reduction in the presence of citrate and an optimized concentration of CTAB (0.1 M) and ascorbic acid (0.1 M) via two–three step growth. In this method, only a small yield with high LSPR AuNRs were obtained. The method was further improved by varying growth solution pH, which resulted in high AR [16]. The addition of various seeds in the growth solution can produce AuNRs with high yield. The surfactant in the growth solution governed the growth of AuNRs by preferential adsorption to the crystallographic faces acting as a soft micellar template [13,16,60]. The addition of reagents such as NaCl, NaNO_3_, or NaBr can also influence the aspect ratio and the yield. The aspect ratio can also be controlled under optimal conditions by varying the seed to HAuCl_4_ ratio. However, increasing the amount of seed solution decreases the AR.

#### 2.4.2. Seed-Mediated Method with AgNO_3_

The introduction of silver nitrate (AgNO_3_) provides better control on AuNRs shapes similar to the electrochemical method but with less energy. In this method, AgNO_3_ is introduced in the growth solution to regulate the gold seeds structural transformation to a rod-like shape; it increases the nanorod yield and passivates crystal facets of AuNRs [65,80]. The low pH of the growth solution controls the reduction of silver ions. Some studies have shown that the aspect ratio of AuNRs can be fine-tuned by adjusting the volume of silver ions in the growth solution while keeping other parameters (such as pH, seed solution addition, etc.) constant. Here, 0.1 M has been reported to be a critical concentration of AgNO_3_ in AuNRs synthesis [65]. By simply adjusting the AgNO_3_ or gold seed volume in the growth solution, the LSPR can be tuned up to 825 nm, which corresponds to an AR of ∼4.5. In addition, the concentration of ascorbic acid can influence the aspect ratio and yield. Alternatively, phenolic and polyphenolic compounds such as hydroquinone [19,81,82], 1,2,3-trihydroxy benzene, 1,2,4-trihydroxy benzene [83], and dopamine [84] have been used as mild reducing agents to replace the ascorbic acid [77]. However, ascorbic acid is still preferred, because the phenolic and polyphenolic compounds’ reduction potentials are lower.

The seed-mediated method with a binary surfactant was first reported by Nikoobakht and El-Sayed in 2003 [70], where they incorporated the use of a binary surfactant in the seed-mediated method. A binary surfactant mixture containing CTAB and benzyldimethylammoniumchloride (BDAC) was used to grow AuNRs of larger aspect ratios (>5). The synthesis of larger aspect ratios AuNRs with the binary surfactants is attributed to its flexible nature compared with the single component surfactant. The CTAB monomers have a larger affinity for the side facets and BDAC monomers bound to the end facets of the rods. This results in faster growth in the longitudinal direction. Additionally, the rigid structure of CTAB monomers maintains one-dimensional growth and also serves to control the reduction of Au^3+^ ions, similar to the mechanism of the seed-mediated without silver ions [70].

Ye et al. [52,53] modified the method by replacing BDAC with sodium oleate (NaOL) and obtained monodisperse AuNRs with broadly tunable dimensions. In addition, they also showed that AuNRs with a high aspect ratio can be synthesized using a bromide-free surfactant mixture [52,53]. Khlebtsov et al. [57] demonstrated a controllable growth of AuNRs in the binary surfactant mixture of CTAB and NaOL. They showed that by controlling the amount of AuNRs addition in the second growth solution, it is possible to tune the average length, diameter, and LSPR peak in a wider range. The functions of NaOL in seed-mediated binary–surfactant synthesis are to serve both as a capping agent and as a reductant, to slow down the reaction, and to control overgrowth [57]. Since the discovery of the seed-mediated method, numerous research groups have continued to develop the synthesis of AuNRs by evaluating the effect of introducing different additives to the growth solution or seed solution such as acetone [58], cyclohexane [58], Na_2_S [85], hydrochloric acid [69], nitric acid [55,56], iodide ions [86,87], bromide ions [88,89], copper ions [90], platinum ions [71], deuterium oxide [91], and small aromatic molecules [68].

### 2.5. Seedless Growth Method

In the seedless method, AuNRs are produced via one-pot synthesis by adding NaBH_4_ directly in the growth solution [54,66,77]. This method is also known as in situ seed formation, and it was initially reported by Jana et al. in 2001 [63]. Similar to the seed-mediated method, different synthetic parameters were evaluated to control the size, aspect ratio, and monodispersity. The nucleation and growth in the seedless method occurs in one single step, with anisotropic growth occurring at the very beginning [66]. Zijlstra at el. [54] reported the effect of temperature in the seedless synthesis of AuNRs. The silver nitrate concentration played a crucial role in producing nanorods at all temperatures. The NRs length decreases with increasing temperature, while the width stays constant throughout the temperature range from 25 to 97 ℃ [54].

Ali et al. [62] reported relatively small monodisperse AuNRs with an average length between 10 and 25 and width between 2.5 and 5 nm, by adjusting the pH and NaBH_4_ concentration of the growth solution. The concentration of silver ions was found to be essential in controlling the aspect ratio of the nanorods [62]. Xu et al. [66] demonstrated that a high aspect ratio of AuNRs with an LSPR larger than 1400 nm can be produced via the seedless method by using paradioxybenzene as a reducing agent. The yield was high without a need for further purification [66]. The use of dopamine as a reductant with low surfactant (CTAB) concentration to produce AuNRs with LSPR ranging from 700 to 1050 nm was reported by Liopo et al. [77]. The size of AuNRs can also be tuned from 7 × 30 nm to 20 × 100 nm. The presence of protonated dopamine enabled a favored interaction with the CTAB bilayer, which led to a high yield in the synthesis of AuNRs (80–95%). The LSPR, size, and aspect ratio were tuned by adjusting the concentration and ratio of silver ions, CTAB and dopamine [77]. Wang et al. [92] used resveratrol as the reducing agent to synthesize AuNRs. AuNRs with a good size monodispersity and a tunable aspect ratio were produced with an LSPR peak ranging from 570 to 950 nm [92].

A binary surfactant mixture of CTAB and NaOL was also used in the seedless method by Lai et al. [59] to produce AuNRs with high yield and better monodispersity [59]. This method was further modified by Roach et al. [61] to achieve high-purity AuNRs with tunable morphology. These were achieved by varying the concentration and ratio of CTAB and NaOL, which enables the formation of monocrystalline AuNRs with diameters between 7 and 35 nm and an aspect ratio ranging from 2.8 to 4.8. The procedure resulted in high monodispersity (size and shape) with good reproducibility and scalability, which allows batch synthesis (500 mL in volume) [61]. Recently, Requejo et al. [67] reported the use of PVP of different molecular weights (5–360 kDa) as an additive for accelerating the synthesis of AuNRs by applying both the seedless and seed-mediated methods. A tunable LSPR wavelength ranging from 700 to 1050 nm was achieved by adjusting the molecular weight of PVP and a time-dependent addition of polymer for both syntheses. For the seedless approach, the growth of AuNRs in the presence of CTAB was accelerated as the molecular weight of PVP increased from 5 to 360 kDa; however, the ARs decreased from 6.69 to 4.20. In the seed-mediated approach, the aspect ratio decreased from 7.41 to 3.72 as the molecular weight of PVP increased to 55 kDa. Both methods revealed that a high molecular weight of PVP caused a reduction in shape uniformity yield, even though smaller AuNRs were produced [67]. PVP primarily functions as a capping or templating ligand to stabilize the growing nanorods, but not as a reducing agent [93]. They compared again the seed-mediated against seedless methods by incorporating thiolated molecules such as glutathione, oxidized glutathione, l-cysteine, and l-methionine during the growth stage. Both methods produced smaller ARs due to the reduction in length and increment in width depending on the method, type of additive, and the strength of its interaction with the NRs surface. The seed-mediated method produced larger sized NRs when oxidized glutathione was used, while the seedless method produced smaller size NRs with high quality using glutathione and oxidized glutathione. The reason behind the high-quality production of AuNRs using thiolated molecules was attributed to the binding strength of thiol, disulfide, and thioether via Au–S interaction, which prevented spherical nanoparticles formation [93,94].

## 3. Graphene Oxide Synthesis

**Figure 3:**
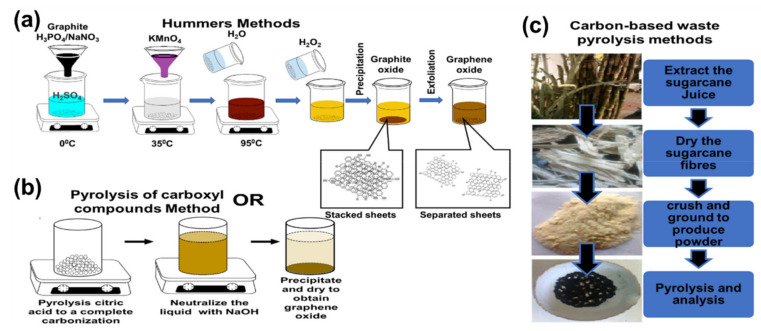
Schematics illustration for different synthesis of graphene oxide (GO): (**a**) Hummers method (**b**) pyrolysis of carboxyl compounds method and (**c**) pyrolysis of carbon-based waste.

Graphene oxide can be synthesized in two ways: bottom–up and top–down approaches. The bottom–up method is mostly time-consuming, and its scalability is not easy. On the other hand, top–down is a widely used method whereby GO is derived from graphene or carbon materials [95]. This method dates back to the middle 1800s by Brodie [96] followed by Staudenmaier [97] and the method of Hummers and Offerman [98], where the first graphitic oxide was proposed. By the early 2000s, the first graphene oxide was reported via the exfoliation of graphite oxide. To date, GO is predominantly prepared using a standard or modified Hummers’ method (Figure 3a) by the prolonged exposure of bulk graphite to strong oxidants such as sulfuric acid, sodium nitrate, and potassium permanganate. The oxidation process also aids the exfoliation of the graphite [23,28,30,95,99,100].

Other synthetic routes such as pyrolysis with the complete carbonization of carboxyl-based compounds, for example, citric acid [101,102] (Figure 3b) and pyrolysis with the complete carbonization of carbon waste materials (e.g., sugarcane fiber waste) (Figure 3c) [103] are also available. The pyrolysis methods are easier and quicker than the Hummers methods; however, this is tricky, because one can easily produce carbon quantum dots (graphene oxide quantum dots/graphene quantum dots) instead of GO during the process. The carbon quantum dots are highly luminescent small-size graphene sheets with a size distribution between 3 and 20 nm. They have unique optical and electronic properties because of their quantum confinement and band edge effects [101,104,105]. Dong et al. [101] reported that a longer heating of citric acid leads to the formation of GO. This has been attributed to the complete carbonization, which forms larger nanosheets with abundant small sp^2^ clusters isolated within the sp^3^ C–O matrix [101]. Wang et al. [105] further reported that the pyrolyzing temperature and heating period play a major role in forming either GO or carbon quantum dots. [105]. Graphene oxide can be chemically reduced to produce less oxygenated graphene, which is called reduced graphene oxide (rGO). However, some of the reducing process/agents conventionally used are harmful to the environment. Currently, there are a number of reports on greener and environmentally friendly reducing agents such as weak acid [106,107,108], amino acids [109,110], and plant extracts [111,112,113,114] for such a reduction.

## 4. Graphene Oxide–Gold Nanorod: Coating and Properties

**Figure 4:**
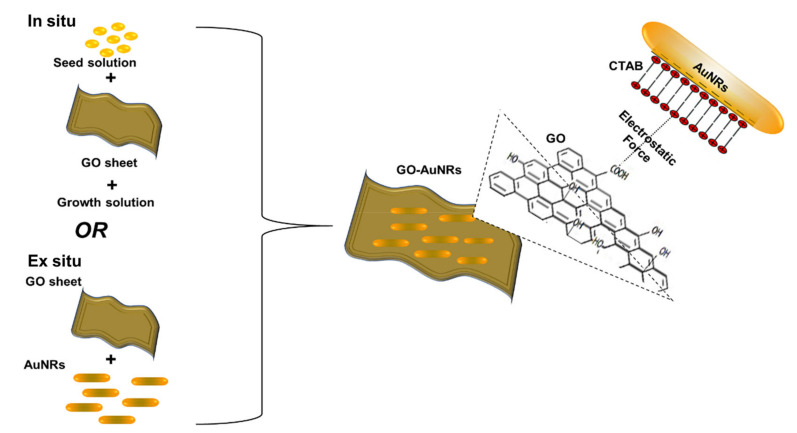
Methods of obtaining GO-coated AuNRs (GO–AuNRs) and the electrostatic bond between AuNRs and GO.

The general two ways of coating AuNRs with GO are usually referred to as in situ and ex situ methods (Figure 4). In situ is the direct growth of AuNRs on the surface of GO, whereas in the ex situ method, both AuNRs and GO are prepared separately and later combined together. The ex situ method can be achieved by a direct electrostatic reaction between GO and AuNRs.

### 4.1. Ex Situ Coating Approach

**Figure 5:**
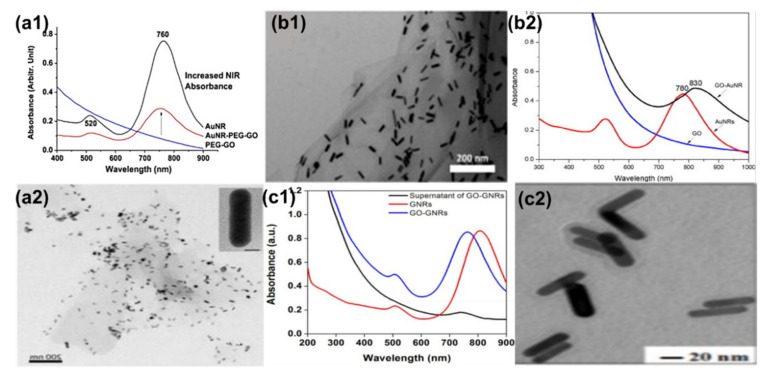
(**a1**) UV-Vis spectrum of the polyethylene glycol (PEG)–GO molecules, AuNRs, and their assembled composites of AuNRs–PEG–GO, (**a2**) TEM images of the AuNRs–PEG–GO composites. An enlargement of the AuNRs–PEG–GO composites is shown for a better demonstration of the assembly (scale bars: 200 nm). The inset figure with the 5 nm scale bar indicates an attached single gold nanorod. Adapted from [29], with permission from Wiley, 2013. (**b1**) TEM image (scale bar: 200 nm) and (**b2**) UV-Vis spectrum of GO–AuNRs Adapted from ref [115], with permission from Elsevier, 2013. (**c1**) UV-Vis spectrum of GO–AuNRs and AuNRs. (**c2**) TEM images of GO–AuNRs (scale bar: 20 nm). Adapted from [118], with permission from RSC advances, 2011.

The ex situ method is widely used for coating AuNRs; however, this direct method leads to the aggregation of AuNRs on the GO sheet [115]. Recently, some studies have used linkers, stabilizers, or functionalization material such as PVP [115], polyethylene glycol (PEG) [29], polystyrene sulfonate (PSS) [38], cysteine (Cys) [116], polyethyleneimine (PEI) [117], and gum arabica (GA) [30] to prevent these aggregations. AuNRs-attached PEG–GO (AuNRs–PEG–GO) nanocomposites by electrostatic interactions were reported by Dembereldorj et al. [29]. The UV-Vis spectrum of AuNRs–PEG–GO showed that the absorbance of the LSPR peak decreased due to the coating with PEG–GO; however, the AuNRs TSPR and LSPR wavelength positions were not affected after modification with PEG–GO. As shown in Figure 5a1, AuNRs were stacked onto PEG–GO molecules due to electrostatic bonding between the AuNRs and PEG–GO (Figure 5(a2)) [29]. Hu et al. [115] used a PVP instead of PEG to stabilize the AuNRs. AuNRs were synthesized using the seed-mediated method, whereas the GO was synthesized using Hummers’ method. Later, GO–AuNRs were synthesized by the electrostatic self-assembly procedure, whereby GO was first mixed with PVP before adding AuNRs in a ratio of 1:5 (AuNRs:GO). In this work, Hu et al. reported that the plasmon coupling between AuNRs adsorbed on the GO surface was responsible for the broadening and red-shifting (from 780 to 830 nm) of the LSPR peak (Figure 5b1). Almost transparent GO sheets perfectly decorated by large amounts of well-dispersed AuNRs were obtained, and the AuNRs were confined in the range of GO sheets (Figure 5b2) [115]. Wei et al. [118] reported a different ex situ approach. They used ammonium hydroxide as a catalyst to wrap GO around AuNRs instead of a stabilizer (Figure 5c1). The LSPR wavelength position blue-shifted from 803 to 760 nm, which implies that the AuNRs aspect ratio was slightly reduced after wrapping with GO nanosheets (Figure 5c2). They further confirmed surface modification using zeta potential, which showed a decrease in surface charge from +25.5 to −18.1 mV due to negatively charged GO sheets [118]. The use of stabilizers with polymers is a promising method for preventing the aggregation of AuNRs upon modification with GO, which is one of the challenges of the ex situ approach.

### 4.2. In Situ Coating Approach

**Figure 6:**
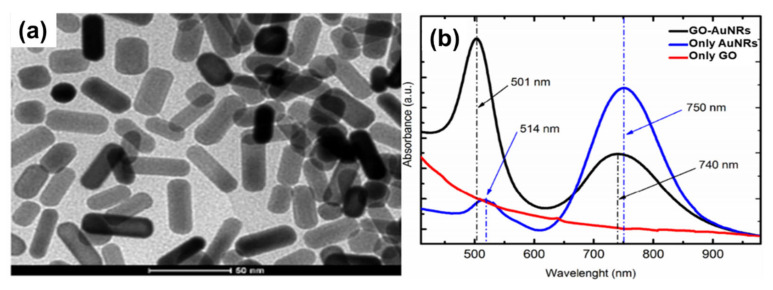
(**a**) TEM images of the GO–AuNRs (scale bars: 50 nm). (**b**) UV-Vis spectrum of the comparison of GO–AuNRs, AuNRs, and GO. Adapted from [119], with permission from RSC advances, 2011.

In situ synthesis is not an as widely used method as ex situ, because it is not easy to control the growth of gold nanorods on the GO surface. Caires and co-workers [119] reported an in situ method where AuNRs were grown directly onto the GO flakes in solution with the help of UV light irradiation. This method was easily scalable; however, it resulted in AuNRs with a low dispersion and aspect ratio (∼3) (Figure 6a). The comparison of the AuNRs and AuNRs grown on the GO flakes showed that both the LSPR and TSPR peaks of GO–AuNRs were blue-shifted from 514 and 750 nm to 501 and 740 nm, respectively, with the increasing TSPR absorbance (Figure 6b). These results were attributed to the strong interaction of the lateral surface of the AuNRs with GO, the change in the local dielectric environment surrounding the surface of the AuNRs, and the increase in polydispersity when the synthesis were performed in the presence of GO [119]. Sun et al. [38] reported the synthesis of GO–AuNRs nanohybrids using the in situ technique, whereby a seed solution was prepared separately and mixed with PSS and GO functionalized with poly(diallyl dimethyl ammonium chloride) (PDDAC) in a growth solution to produce GO–AuNRs. They compared this in situ method of GO–AuNRs with an ex situ method, and they reported that the in situ method was able to address the issue of the aggregation of the AuNRs in GO sheets, which is common in the ex situ approach [38]. Khan et al. [30] reported the in situ preparation of GO–AuNRs, whereby gum arabic (GA) acted as both a reducing agent and stabilizer. Initially, GO was functionalized with GA and was added to the AuNRs growth solution before adding seed solution. A comparison of the UV-Vis spectra of AuNRs and GO–AuNRs showed a blue-shifted peak in both the TSPR and LSPR of GO-AuNRs. This has been attributed to the alterations in the refractive indices of AuNRs surface as well as the surrounding medium. AuNRs with bone-shaped edges were obtained, and this was attributed to the competency of GO with both the CTAB and Ag^+^ ions during the growth process [30].

## 5. Biocompatibility

**Figure 7:**
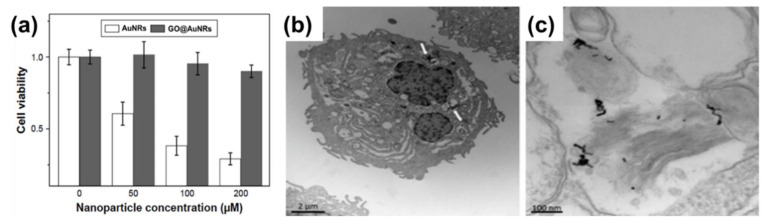
(**a**) Cell viabilities of AuNRs and GO@AuNRs against A549 cells. Reproduced from [37], with permission from Dove Medical Press, 2017. (**b**,**c**) High-magnification transmission electron microscopy image of internalization of AuNRs–PEG–GO composite in a cells. Adapted from [29], with permission from Wiley, 2013.

The biocompatibility of all nanomaterials is very essential, especially if the materials are to be used for biological application. Numerous studies on nanomaterials’ cytotoxicity have shown that the size, surface charge, and capping agents are mostly responsible for the toxicity found in nanomaterials [120,121,122,123]. AuNRs are mostly synthesized with CTAB as a surfactant, which causes the toxicity of the material [12,17]. In contrast, GO has been shown to be biocompatible, and the combination of AuNRs and GO has been shown to produce a more biocompatible composite compared to unmodified AuNRs. In a recent development, Qiu et al. [37] evaluated the cytotoxicity of GO@AuNRs against adenocarcinomic human alveolar basal epithelial cells (A549 cells) (Figure 7a). They observed that GO@AuNRs were more biocompatible even at 200 μM than bare AuNRs [37]. In another study, gold nanorod-decorated (AuNRs–PEG–GO) nanocomposites were reported to be biocompatible when their cytotoxicity was evaluated against epidermoid carcinoma cells (A431 cells). Cellular internalization from dark-field microscopy, confocal Raman microscopy, and transmission electron microscopy (TEM) confirmed that AuNRs–PEG–GO did not affect concentrations up to 1 ppm (Figure 7b,c) [29].

**Figure 8:**
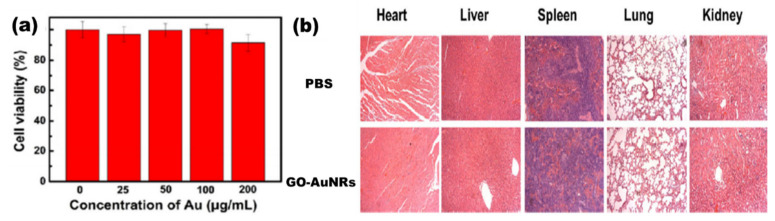
(**a**) Cytotoxicity of GO–PSS–AuNRs solutions with various Au concentrations against SW1990 cancer cells. (**b**) Histological images of the heart, liver, spleen, lung, and kidney of the mice, obtained one month after intravenous injection with phosphate buffer solution (PBS, 100 μL) and GO–AuNRs (0.026 M, 100 μL). Adapted from [38], with permission from Springer Nature, 2016.

Lim et al. [31] reported the synthesis of plasmonic gold nanoshells (AuNSs) and AuNRs coated with rGO and evaluated their cytotoxicity against human umbilical vein endothelial cells (HUVECs). HUVECs exposed to GO-coated particles for 24 h at an optical density of 1.0 had no significant cytotoxicity [31]. Another study by Sun et al. [38] reported the cell viability of the human pancreatic adenocarcinoma cell line (SW1990 cancer cells) against GO–PSS–AuNRs (Figure 8a). Different concentrations of GO–PSS–AuNRs nanohybrids (0–200 μg/mL) exposed to the cells for 24 h were measured by a Cell Counting Kit-8 (CCK-8) assay, and all concentration tested showed no significant toxicity, even at high concentration (200 µg/mL). The in vivo long-term toxicity assessment of the GO–AuNRs after a month of exposure did not show obvious inflammation, cell necrosis, or apoptosis to normal organs (Figure 8b) [38].

## 6. Photothermal Properties

**Figure 9:**
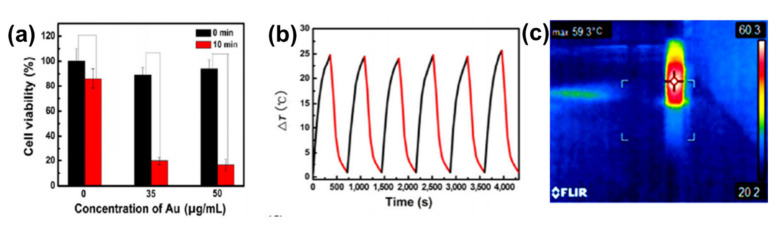
(**a**) Phototoxicity assay of SW1990 cells in the presence of different amounts of GO–PSS–AuNRs with or without laser irradiation (808 nm) for 10 min. (**b**) Photothermal profiling of on and off cycle of GO–AuNRs (50 µg/mL) for 4000 s. Adapted from [38], with permission from Springer Nature, 2016. (**c**) IR image of GO–AuNRs irradiated with laser. Adapted from [30] with permission from Elsevier, 2017.

The photothermal properties of AuNRs and GO have been researched widely as individual entities and both AuNRs and GO have shown remarkable in vitro and in vivo photothermal efficiency [29,30,34,35,38,115,117,124,125]. However, GO needs to be reduced to have these properties, because GO has a highly oxidized structure with a disrupted π conjugation, which lowers its conductivity [30,33]. The photothermal property of AuNRs is due to its surface plasmonic resonance, and as mentioned earlier, AuNRs have two types of surface plasmonic resonance peaks [3,126,127,128,129,130]. Numerous studies have shown that AuNRs can generate much heat when exposed to a specific laser with a wavelength corresponding to its LSPR [1,3,6]. The combination of AuNRs and GO for effective photothermal properties has been studied [29,30,31,32,34,35,36,39,125]. Dembereldor et al. [29] investigated the photothermal effect of the AuNRs–PEG–GO nanocomposites against A431 cells. The AuNRs–PEG–GO was found to produce heat when exposed to a laser with a 60 W/cm^2^ for 5 min. This nanocomposite was able to significantly destroy the cells by ≈40% when irradiated with light. [29]. Lim and co-workers [31] reported a comparative study of AuNRs and gold nanoshells (AuNSs) coated with rGO. rGO–AuNRs showed outstanding photothermal property compared to the uncoated AuNRs, AuNSs, and rGO–AuNSs [31]. Sun et al. [38] reported that the GO–PSS–AuNRs displayed an outstanding photothermal effect in vitro (Figure 9a). The temperature of the GO–PSS–AuNRs nanohybrids increased from 25 to 49.9 °C at a concentration of 50 μg/mL after irradiation with an 808-nm laser (0.4 W/cm^2^) for 6 min (Figure 9b). In addition, GO–PSS–AuNRs exhibited good optical and morphological stability and photothermal properties, even after six cycles of laser irradiation [38]. Khan et al. [30] prepared GO@AuNRs by functionalizing natural polymer gum arabic to reduce GO, which was further conjugated to AuNRs to increase its photothermal profiling. An infrared camera was able to detect up to 59.3 ℃ heat produced by GO–AuNRs (Figure 9c) [30]. In another development, Turcheniuk et al. [35] investigated the potential of PEG-functionalized rGO–PEG enrobed AuNRs conjugated to Tat protein for the photothermal destruction of human glioblastoma astrocytoma (U87MG) cells in mice. The Tat protein was used to make the composite target selective. In vivo studies showed that the composite was able to suppress U87MG tumor growth in mice [35].

## 7. Applications

### 7.1. Biomedical Application: Theranostic Agent

**Figure 10:**
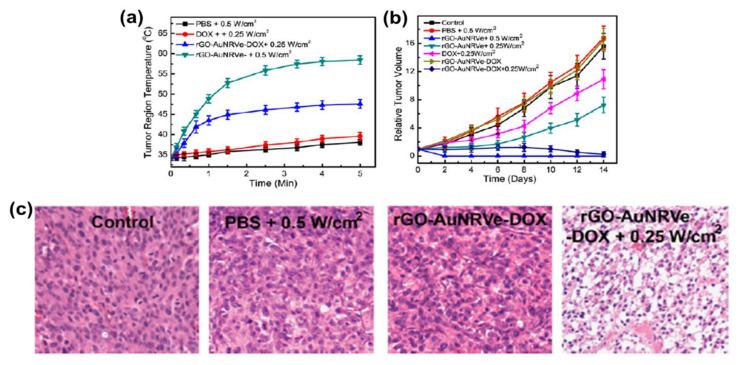
(**a**) Tumor region temperature changes of the mice treated with PBS, DOX, and rGO–AuNRsVe–DOX irradiated with an 808 nm laser of different power densities for 5 min. (**b**) Relative tumor volume of the tumor-bearing mice after intravenous injection of the samples and exposed to the 808 nm laser at different power densities. Tumor volumes were normalized to their initial sizes. (**c**) Images of H&E (hematoxylin and eosin)-stained tumor sections harvested from the tumor-bearing mice treated with PBS and rGO–AuNRsVe–DOX with or without laser irradiation. Adapted from [34] with permission from American Chemical Society, 2015. Ve: vesicle.

The combination of therapy and diagnosis that is also known as theranostic is becoming one of the most interesting studies in cancer research [131] due to its great potential in personalized cancer medicine. The theranostic agent must have a characteristic such as the ability to diagnose a disease, its status, and its response to a specific treatment while at the same time perform treatment [132]. The coating of AuNRs with GO has produced a material that is biocompatible and useful in biomedical application. However, studies of GO-coated AuNRs as a theranostic agent are still rare in cancer therapy. The most common studies of GO–AuNRs are based on conjugation with a cancer drug. Song et al. [34] fabricated an rGO–AuNRs vesicle (Ve) with remarkably amplified photoacoustic (PA) performance and photothermal effects when loaded with doxorubicin (DOX). Both the cavity of the vesicle and the large surface area of the encapsulated rGO can be used for loading DOX, making it an excellent drug carrier. The combination of chemo- and photothermal therapies revealed an effective inhibition of tumors. The in vivo studies of rGO–AuNRsVe–DOX generated more heat even at low power density than bare rGO–AuNRsVe (Figure 10a). The tumor-bearing mice were intravenously injected with PBS, DOX, and rGO–AuNRsVe–DOX and exposed to the 808 nm laser at different power densities (Figure 10b). The relative tumor volume was reduced when treated with rGO–AuNRsVe–DOX irradiated at lower power density compared with bare rGO–AuNRsVe irradiated at higher power density. The tumor tissue section under treatment with rGO–AuNRsVe–DOX irradiated with laser showed more severe cancer necrosis and fewer cancer cells (Figure 10c). On the contrary, mice treated with PBS, laser irradiation, or rGO–AuNRsVe–DOX without laser irradiation did not exhibit any tumor necrosis [34].

**Figure 11:**
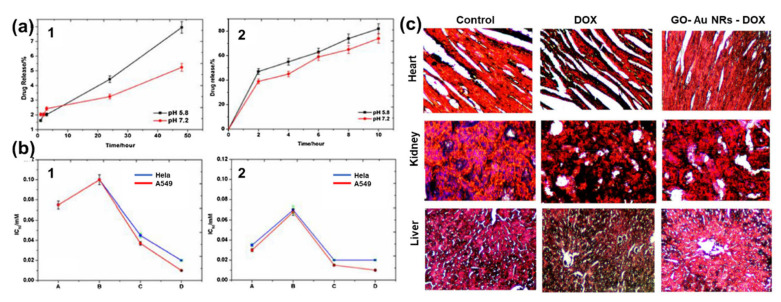
(**a**) Percentage of drug release with respect to time (**1**) without near-infrared (NIR) and (**2**) with NIR irradiation. (**b**) IC_50_ values on in vitro cell lines (**1**) without NIR irradiation and (**2**) with NIR irradiation. (**c**) Histological images of a heart, kidney, and liver treated by DOX and fGO–AuNR–DOX Adapted from [30], with permission from Elsevier, 2017.

Khan et al. [30] also reported DOX conjugated to GO–AuNRs, however, with a little modification whereby GO was first functionalized with GA before coating with AuNRs and conjugation with DOX. The drug release kinetics under physiological conditions using standard statistical models demonstrated that at pH 5.8, 82% of DOX from GO–AuNRs–DOX was released under laser irradiation (Figure 11a). GO–AuNRs were shown to have low dark toxicity in both Hela and A549 cells; however, when conjugated to DOX, a significant decrease in cell viability occurred, and there was even more when the laser was applied (Figure 11b). The in vivo experiment results indicated that when mice were treated by only DOX, it caused liver and kidney tissue damage (Figure 11c). In contrast to DOX, the GO–AuNRs–DOX showed no damage to the organs. The tissue damage by DOX alone was believed to be due to the high uptake of the drug; however, the biocompatibility of GA used on the complex and slow release of DOX was reported to be the reason, while no organ was damaged, even after irradiation [30].

Xu et al. [36] demonstrated that AuNRs encapsulated in GO decreased the toxicity of the surfactant-coated AuNRs and offered high surface area for the conjugation of hyaluronic acid. The composite was used to load DOX, which was demonstrated to be more effective than chemo- and photothermal therapy when used separately [36]. Zang et al. [39] introduced mesoporous silica on an rGO–AuNRs composite (Au/SiO_2_/rGO) and also conjugated it to DOX. Au/SiO_2_/rGO nanohybrids exhibited photothermal stability and good drug release. [39]. Qi et al. recently reported the immobilization of AuNRs onto the surface of GO–PEG via polydopamine (PDA) to fabricate AuNRs/GO@PDA hybrid nanosheets. The AuNRs/GO@PDA hybrid nanosheets were photostable and biocompatible with a photothermal conversion efficiency of 14.1%, which was higher than the bare AuNRs. In addition, AuNRs/GO@PDA was an efficient drug carrier that possessed a loading ability for DOX of up to 86.16% [133]. More studies using GO-coated AuNRs still need to be conducted to advance its usefulness as a theranostic agent.

### 7.2. Sensors

**Figure 12:**
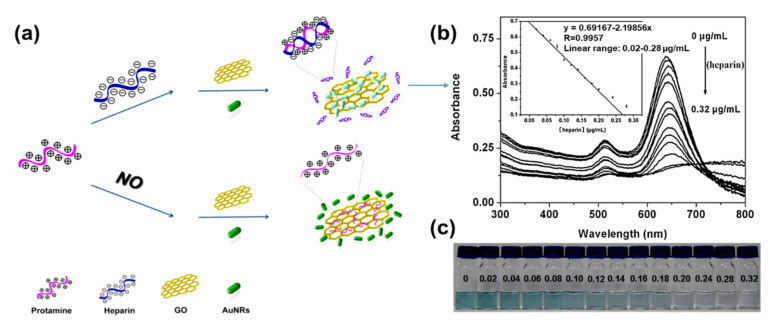
(**a**) Schematic of GO–AuNRs protamine mixed sensor for detecting heparin. (**b**) Absorption spectra of GO–AuNRs protamine mixed solution with difference heparin concentrations. Inset: heparin detection calibration curve (**c**) Photographic images of the corresponding colorimetric responses with the increase of heparin concentration. Adapted from [43], with permission from Elsevier, 2012.

The application of GO–AuNRs as an analytical sensor has gained more interest due to their unique properties of both AuNRs and GO. The surface plasmon resonance (SPR) of AuNRs is one of the reasons why it has emerged as an excellent tool for sensing applications [76], while the GO with various oxygen functional groups can also act as a molecular sensor [134]. The integration of AuNRs-decorated GO have been shown to enhance the sensing performance [5,40,41,44,45,116,135,136,137]. Both of these materials have been used for various applications from biosensors to chemosensors, for the detection of DNA, pollutants, diseases, and drugs, to mention a few. Fu et al. [43] developed GO–AuNRs for the detection of heparin by applying the color-quenching capacity of GO. Briefly, AuNRs were self-assembled onto the surface of GO through electrostatic interaction (Figure 12a), which decreased the LSPR as well as its appearance. The original color was restored by adding polycationic protamine due to its strong interaction with GO and its strong affinity for heparin over GO. The sensor experiments were carried out in a 4- (2-Hydroxyethyl)-1-piperazineethanesulfonic acid buffer (pH 7.4, 10 mM) at room temperature. The result showed a linear relationship to heparin concentration, ranging from 0.02 to 0.28 μg/mL (R = 0.9957) with a detection limit of 5 ng/mL (Figure 12b). Figure 12c shows the photographic images of the corresponding colorimetric responses with the increase of heparin concentration [43].

In another development, Zhang and co-workers [138] reported an SPR-based biosensor for the detection of transferrin using GO decorated with AuNRs–antibody conjugates carried out in a reactor using phosphate buffer solution (PBS) as the baseline solution. In this study, AuNRs were anchored on the surface of GO with the antibody (rabbit anti-transferrin), which served as an enhancer for the detection of transferrin. The AuNRs–GO antibody conjugate biosensor showed a detection response to transferrin in the concentration range of 0.0375–40 μg/mL [138]. Deng et al. [135] also reported the coating of a glassy carbon electrode (GCE) with AuNRs-decorated GO nanosheets. The AuNRs facilitated the electrochemical reduction of GO. The electrochemically reduced graphene oxide decorated with AuNRs demonstrated high accumulation efficiency and considerable surface enhancement effects for the electro-oxidation of sunset yellow and tartrazine. The probe had a linear response to sunset yellow and tartrazine in the concentration range of 0.01–3.0 μM and 0.03–6.0 μM with detection limits of 2.4 and 8.6 nM, respectively [135]. Arvand and Gholizadeh [40] investigated an AuNRs–GO nanocomposite incorporated carbon nanotube paste-modified glassy carbon electrode. The square wave voltammetry electrochemical method was used for the determination of indomethacin in aqueous media (PBS, pH 8.0). This probe demonstrated a high effective surface area, more reactive sites, and excellent electrochemical catalytic activity toward the oxidation of indomethacin with two linear calibration ranges of 0.2–0.9 and 2.5–91.5 μM and exhibited an excellent limit of detection of 1.7 × 10^−2^ μM. The sensor was further used to detect indomethacin in pharmaceutical samples (tablet and capsules), human blood serum, and urine obtained from the patients who underwent treatment with indomethacin. The accuracy of determination was not different from the labeled values on the pharmaceutical samples by more than 2.56%. The recovery of indomethacin from the blood and urine was reported to be from 98.0% to 103.5%, respectively [40].

**Figure 13:**
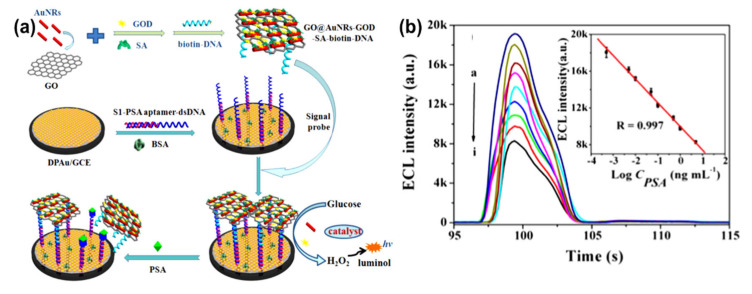
(**a**) Schematic diagram of GO–AuNRs multi-labeled with glucose oxidase and streptavidin toward luminol-based electrochemiluminescence (ECL) aptasensor for prostate-specific antigen detection. (**b**) Electrochemiluminescence intensity changes with prostate-specific antigen (PSA) concentrations (ng/mL), insert: The detection of prostate-specific antigen calibration curve. Adapted from [42], with permission from Elsevier, 2018.

AuNRs-decorated GO sheets have also been used as a simple electrochemical sensor for sensitive and selective DNA detection. Differential pulse voltammetry (DPV) was used to monitor the DNA hybridization occurrence with methylene blue as an electrochemical indicator. The methylene blue peak currents were linear with the logarithm of the concentrations of complementary DNA from 1.0 × 10^−9^ to 1.0 × 10^−14^ M, and the detection limit of the probe was measured to be 3.5 × 10^−15^ M. Furthermore, this electrochemical sensor probe can distinguish complementary DNA sequences in the presence of a large amount of single-base mismatched DNA (1000:1), indicating that the biosensor has high selectivity [44]. Similarly, an electrochemical biosensor for the detection of specific-sequence target DNA has been reported by Shi et al. [137]. The biosensor was fabricated based on a “sandwich-type” detection strategy, which involved a capture probe immobilized on the surface of the AuNRs-decorated rGO sheets, gold nanoparticles as a reporter probe to flank the target DNA, and adriamycin. Adriamycin was used as an electrochemical indicator because of its ability to electrostatically bond with anionic phosphate of DNA strands. The peak currents of adriamycin in DPV were linear with the logarithm of target DNA concentration in the range of 1.0 × 10^−16^ to 1.0 × 10^−9^ M. The probe had a detection limit of 3.5 × 10^−17^ M. This sandwich-type sensor exhibited good selectivity, even for single-base mismatched target DNA detection. In another study by Azimzadeh et al. [41], GCE modified with a thiolated probe-functionalized AuNRs-decorated GO sheet was used to detect a different nucleic acid (miRNA), using oracet blue as an indicator in a DPV. In addition, AuNRs–GO exhibited high specificity, with the ability to differentiate between complementary target miRNA, single-, three-base mismatch, and non-complementary miRNA. The GCE modified with thiolated-functionalized GO–AuNRs has an electrochemical signal (peak current) linear relationship with the concentration of the target miRNA ranging from 2.0 fM to 8.0 pM and a detection limit of 0.6 fM. All electrochemical sensing was carried out in a phosphate buffer solution (pH 7.0) [41]. Cao et al. [42] constructed GO–AuNRs multi-labeled with glucose oxidase (GOD) and streptavidin (SA) to form a luminol-based electrochemiluminescence (ECL) aptasensor for detecting prostate-specific antigen (PSA) (Figure 13a). To achieve multiple signal amplification, GOD and SA–biotin–DNA were deposited on GO–AuNRs to form a signal probe. The addition of glucose produced H_2_O_2_ due to the catalytic effect of GOD incorporated on the probe, while the AuNRs reacted with the H_2_O_2_ to produce reactive oxygen species in a luminol ECL reaction. The combination of SA with biotin–DNA was used to intensify the ECL signal intensity. The addition of PSA interfered with the interaction between signal amplifiers and the electrode by attaching to PSA aptamers to block/cleave the signal amplifiers, which caused the loss of the ECL signal, as shown in Figure 13b. This ECL biosensor had a linear range of 0.5 pg/mL to 5.0 ng/mL with the detection limit of 0.17 pg/mL (S/N = 3). The ECL biosensor was further used to detect PSA in human serum samples; the recoveries were between 81.4% and 116.0%, with a relative standard deviation varying from 2.8% to 10.2% [42].

Another ECL aptasensor based on a dual-potential signal amplification strategy triggered by graphene/hemin/gold nanorods/G-quadruplex–hemin (rGO–H–AuNRs–G4H) composite was reported by Govindaraju et al. [74]. The rGO–H–AuNRs–G4H showed a good linear detection of thrombin ranging from 100 ng/mL to 0.5 pg/mL linearly, with a detection limit of 4.2 fg/mL [48]. Jayabal et al. [45] reported reduced graphene oxide–AuNRs embedded in an amine-functionalized silicate sol–gel matrix (rGO–Au–TPDT NRs) composite for the electrochemical sensing of nitric oxide (NO). The mechanism of this probe was based on the oxidation of NO by the synergistic catalytic effect of the composite material. The amperometric current of this probe exhibited a linear relationship with the NO concentration ranging from 10 to 140 nM. The probe had a detection limit of ≈6.5 nM. Nirala et al. [136] proposed a bioelectrode from partially reduced GO–AuNRs reinforced with chitosan fabricated on Indium Tin Oxide (CH–prGO–AuNRs/ITO) for glucose detection. These CH–prGO–AuNRs/ITO bioelectrodes showed a high sensitivity of current 3.2 µA/(mg/dL)/cm^2^. CH–prGO–AuNRs exhibited a linear range of 25–200 mg/dL with a low detection limit of 14.5 mg/dL. Furthermore, these CH–prGO–AuNRs/ITO-based electrodes demonstrated synergistically enhanced sensing properties when compared to simple graphene oxide-based CH–GO/ITO electrode. Liu et al. [139] presented a different approach by applying the surface-enhanced Raman spectroscopy (SERS) method with GO–AuNRs as a probe for the detection of hepatitis B surface antigen (HBsAg). This biosensor showed a SERS signal that increased as the HBsAg concentration increased from 1 to 1000 pg/mL with a detection limit of 50 × 10^−3^ pg/mL. They further used the sensor probe to detect hepatitis B in human blood samples from nine infected patients, the detection relative standard deviation was less than ±5%, and the recovery was 96–104% [139]. Lu et al. [140] reported a different approach, where bovine serum albumin-coated silver indium sulfide quantum dots (BSA-AIS) were used as photoelectrochemical (PEC) sensors fabricated with AuNRs and GO for the detection of dopamine (DA). GO and AuNRs were used to enhance the catalytic and photoelectric properties of the sensors. The BSA-AIS/AuNRs/GO-based PEC probe showed a sensitivity in the linear range of 0.3–10 μM and detection limit of 66.8 nM. The same probe was shown to be selective for DA detection over the interfering substances of ascorbic acid and uric acid [140].

### 7.3. Other Applications

GO–AuNRs have also been applied in antibacterial, photoacoustic, and SERS bioimaging application. Turcheniuk and co-workers developed non-antibiotic-based treatments against bacterial infections by Gram-negative apathogenic. Their report illustrated that AuNRs coated with rGO–PEG (rGO–PEG–AuNRs) functionalized with multimeric heptyl α-D-mannoside can selectively kill uropathogenic Escherichia coli UTI89 bacteria causing urinary infection [125]. In another work, Moon et al. reported the photoacoustic (PA) performance of rGO–AuNRs. Simulation results revealed that rGO–AuNRs can generate a higher magnitude of the enhanced electromagnetic field, which is a promising deep-tissue imaging probe due to remarkably high PA amplitudes [32]. Qiu et al. investigated GO–AuNRs for ultrafast NIR SERS bioimaging. The GO–AuNRs in vitro study indicated that it had a higher NIR SERS activity in comparison to traditional gold nanostructures. Hence, it can be a promising probe for NIR SERS-based bioimaging applications [37].

## 8. Conclusions, Remarks, and Future Prospects

In conclusion, the application of GO–AuNRs composites is growing continuously. A comprehensive discussion on GO–AuNRs composite synthesis and various applications were highlighted in this review. The AuNRs synthesis approaches such as hard template method, electrochemical method, photochemical method, seed-mediated method, and lastly the seedless method were discussed. In addition, the coating of AuNRs with GO was reviewed by looking at the interaction of the two materials and how it has been improved to reduce the aggregation of AuNRs on the GO sheet. The coating approaches such as ex situ and in situ, the uses of linkers, stabilizers, or functionalization materials were also reviewed. The cytotoxicity of GO–AuNRs were further discussed, which revealed the biocompatibility of GO–AuNRs composite in both cell and animal studies. The photothermal properties of GO–AuNRs from different reports showed that GO is not only making AuNRs biocompatible but also increasing its photothermal properties. Furthermore, different applications of GO–AuNRs in photothermal therapy, theranostics, sensors, as an antibacterial agent, photoacoustic, and SERS bioimaging were also discussed. Although AuNRs–GO composites have been useful for many applications, some issues such as the weak interaction of AuNRs with GO, which leads to the aggregation and the non-uniform distribution of AuNRs on the GO sheet, still need to be addressed. In addition, the mechanism behind the peak shifting when the stabilizer is used still need to be investigated. In addition, the biological application of GO–AuNRs still needs further investigation. The toxicity of this composite needs to be deeply understood by studying the mechanism of interaction between the cell and the composite, as well as the cellular uptake. Furthermore, the biosensor of this composite needs to be investigated more in real samples.
